# Formalizing psychological interventions through network control theory

**DOI:** 10.1038/s41598-023-40648-x

**Published:** 2023-08-24

**Authors:** Julia Elina Stocker, Georgia Koppe, Hanna Reich, Saeideh Heshmati, Sarah Kittel-Schneider, Stefan G. Hofmann, Tim Hahn, Han L. J. van der Maas, Lourens Waldorp, Hamidreza Jamalabadi

**Affiliations:** 1 Department of Psychiatry and Psychotherapy, Philipps University of Marburg, Rudolf-Bultmann-Straße 8, 35039 Marburg, Germany; 2 Department of Theoretical Neuroscience, Medical Faculty Mannheim, Central Institute of Mental Health, Heidelberg University, Mannheim, Germany; 3 Department of Psychiatry and Psychotherapy, Medical Faculty, Central Institute of Mental Health, Heidelberg University, Mannheim, Heidelberg, Germany; 4 German Depression Foundation, Leipzig, Germany; 5 Depression Research Center of the German Depression Foundation, Department for Psychiatry, Psychosomatics and Psychotherapy, Goethe University, Frankfurt, Germany; 6 Department of Psychology, Claremont Graduate University, Claremont, CA USA; 7 Department of Psychiatry, Psychotherapy and Psychosomatic Medicine, University Hospital of Würzburg, Würzburg, Germany; 8 National Center of Affective Disorders, Würzburg, Germany; 9 Department of Psychiatry, University College of Cork, Cork, Ireland; 10 Department of Psychiatry and Neurobehavioural Science, University College Cork, Cork, Irland; 11 Department of Psychology, Philipps University of Marburg, Marburg, Germany; 12 Institute for Translational Psychiatry, University of Münster, Münster, Germany; 13 Psychological Methods Group, University of Amsterdam, Amsterdam, The Netherlands; 14 National Center of Affective Disorders, Marburg, Germany

**Keywords:** Psychology, Computational science

## Abstract

Despite the growing deployment of network representation to comprehend psychological phenomena, the question of whether and how networks can effectively describe the effects of psychological interventions remains elusive. Network control theory, the engineering study of networked interventions, has recently emerged as a viable methodology to characterize and guide interventions. However, there is a scarcity of empirical studies testing the extent to which it can be useful within a psychological context. In this paper, we investigate a representative psychological intervention experiment, use network control theory to model the intervention and predict its effect. Using this data, we showed that: (1) the observed psychological effect, in terms of sensitivity and specificity, relates to the regional network control theoretic metrics (average and modal controllability), (2) the size of change following intervention negatively correlates with a whole-network topology that quantifies the “ease” of change as described by control theory (control energy), and (3) responses after intervention can be predicted based on formal results from control theory. These insights assert that network control theory has significant potential as a tool for investigating psychological interventions. Drawing on this specific example and the overarching framework of network control theory, we further elaborate on the conceptualization of psychological interventions, methodological considerations, and future directions in this burgeoning field.

## Introduction

Networks are increasingly being utilized in psychological sciences to model complex psychological behaviors in relation to, and as a result of, interactions between psychological components^1,2^. A psychological network is defined by nodes, which are identified with variables observed within a certain context (e.g., clinical symptoms of depression) and their connections, which indicate their interactions^2,3^ e.g., rumination in relation with sleep quality. Such a simple conceptualization of psychological behavior has proven generative, advancing the field in several key areas. Examples include, among others, the study of mental disorders in terms of networks of symptoms^3^, human interactions in social psychology^1,4–7^, and cognitive sciences^8^. Future applications could involve the prediction of relapses of mental disorders as well as contribute to developing novel psychotherapeutic interventions^9^.

Yet, the network approach as currently used has a major limitation: networks are commonly modelled as static constructs i.e., they present a fixed representation of the psychological behavior. Consequently, networks often fail to formalize “how much” the psychological variables change as a consequence of the interactions and external perturbations. Within a clinical case study, for example, the network approach offers insights into whether different symptoms are interrelated (e.g., rumination and sleep quality). However, it does not directly relate the “size” of change in one component (e.g., rumination) to the “size” of change in other variables (e.g., sleep quality). Examples like this are numerous and include virtually any study that contains an intervention such as controlled experiments with more than one condition^10^.

**Figure 1:**
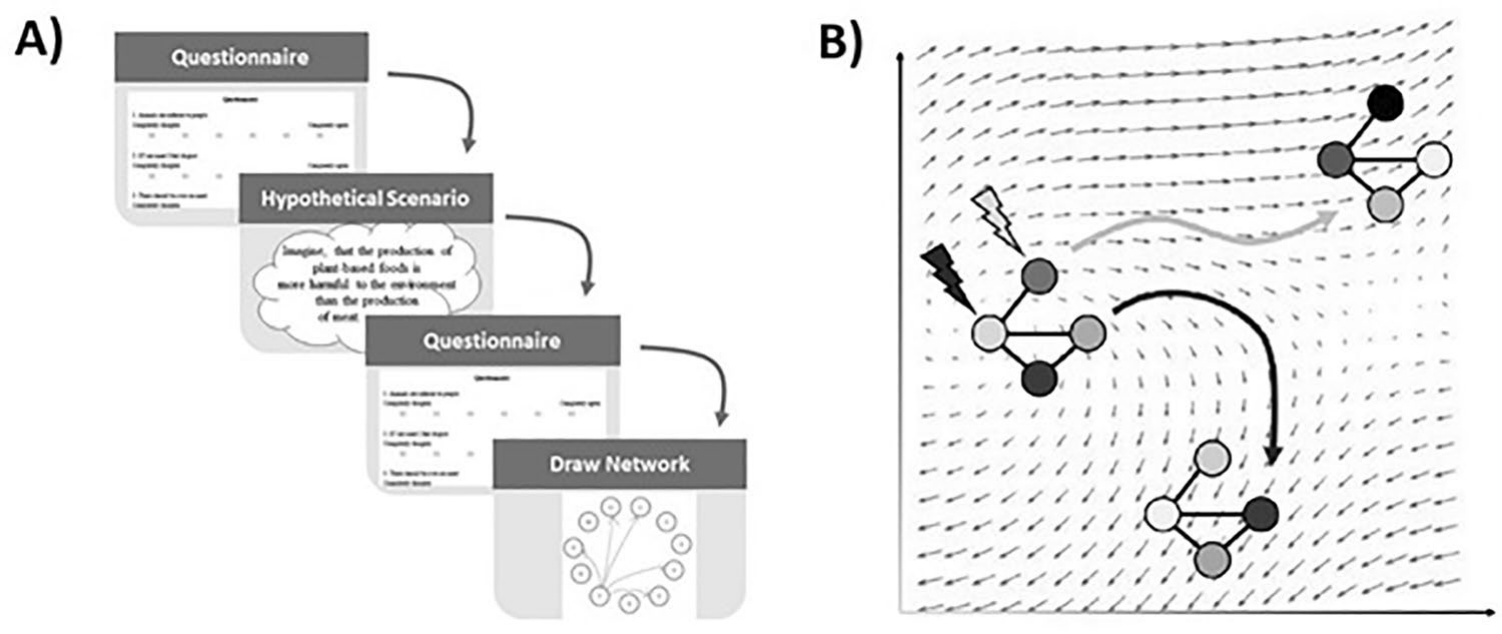
A schematic view of the data and network control theory approach to quantify and predict the effect of interventions. (**A**) Thirty participants answered eleven questions about their attitude towards the consumption of meat. Based on their responses, they are asked to contemplate certain scenarios that are designed to alter their opinion. After this “intervention”, the participants are once more asked the same questions and asked to draw a schematic of connections between the items. (**B**) The effect of interventions in the context of psychological networks can be understood in terms of network control theory. Each network structure dictates the possible transitions of network values (illustrated here in terms of the arrows). The geometry of these arrows relates to the network structure and the dynamic imposed on them and can be linear or nonlinear. Figure 1A adapted from Hoekstra et al^35^.

Importantly, this and similar questions have been systematically addressed in the engineering context^11^. Specifically, dynamical systems theory concerns how the interactions between the components in a network result in a complex behavior^12^. And network control theory, a subset of dynamical systems theory, provides a mathematical foundation to relate observations (i.e., sleep quality) and interventions (i.e., experimental condition)^13,14^. Within this framework, a psychological intervention is considered as any external stimulus (e.g., exposure to a task, medication, therapy, etc.) or alteration in conditions (e.g., change in the task parameters) that might influence the psychological construct being studied. The effect of such interventions is conceptualized as a temporal cascade of minor changes to the network variables (i.e., nodes, see Fig. 1 for a schematic example). From a conceptual standpoint, these models are generative; they mimic the behavior of network variables, such as the components of the specific psychological construct under investigation, as they respond to different conditions. For example, given specific starting conditions, such as the present values of clinical symptoms of major depression, and a range of potential interventions such as sleep deprivations, these models offer a quantitative viewpoint to understand how the symptoms evolve (see Jamalabadi et al.^15^ for an example). In a similar vein, network control theory provides a framework of formulations to comprehend the “behavior” of these models and thus the phenomena they mathematically represent e.g. the clinical symptoms. By adhering to the methodology detailed within network control theory, at least three specific theoretical results can be inferred. First, network control theory facilitates a systematic theory-driven assessment of the general difficulty in inducing changes in the whole network (e.g., all symptoms) following alterations in a specific variable. Significantly, for estimating this category of measures, known as the "controllability," one does not require the precise details of the alteration to the target variable (details outlined formally in Sect. 4.3). Second, an estimation can be made regarding the overall challenge encountered when the network's activity changes, or is intended to change, across various potential conditions. This estimation is quantified as the total "energy" and remains applicable even when the exact intervention is unspecified. Third, one could assimilate the whole temporal evolution of psychological behavior based on the psychological network and thus, predict further hypothetical intervention effects, potentially leading to novel intervention targets (see Lunansky et al.^9^ for a discussion on simulation based intervention design).

If control theory is going to inform interventions in psychological sciences, however, it must first be examined if and how the engineering concepts on which control theory is founded translate into the context of psychological networks. At a fundamental level, the idea is that an intervention, such as therapy or medication, influences various elements within a network, like thoughts or behaviors, in a manner that is proportional to both the intensity of the intervention and the of those elements (so-called locally linearizable assumption^11^). Across engineering domains and more recently neurosciences, this fundamental concept has made analytical treatment of observed phenomena possible and has stimulated progress in various directions such as understanding the human brain under a wide range of neural stimulation^16–19^.

Within the realm of psychology, progress has been somewhat gradual, primarily emphasizing the application of dynamical systems' mathematical framework to better "characterize" observed phenomena. Yet, the current reports are encouraging. For instance, Hilbert and Marchand^20^, within the context of educational psychology, discuss the potential role of dynamical systems in aligning theory, model and data. Within clinical psychology and the closely related psychiatric community, an increasing number of scientists are calling to use the dynamical systems approach to better understand the course of mental disorders^3,15,21–23^. Simulation studies using this approach in studying complicated grief^24^, Post-Traumatic Stress Disorder (PTSD)^9^, and panic disorder^25^ yield strongly consistent results with what is known from the literature. Recently, studies by Hahn et al.^26^ and Jamalabadi et al.^15^, leveraging longitudinal measurements from mobile phones, have indicated that depressive symptom fluctuations align with predictions from network control theory. Applied to Ecological Momentary Interventions (EMI), Fechtelpeter et al.^27^ showed that the results from network control theory can provide insightful information on putative mechanisms of change. Further, network control theory has been used to study the brain-behavior constructs ranging from studies in the clinical setting such as depression^28,29^, to cognitive concepts such as creativity^30^, and further to conceptual frameworks applied to psychological well-being^31^, clinical psychology^24^, and networked systems^9,32^.

Yet, these studies did not probe interventions (with the exception of Fechtelpeter et al.^27^). Subsequent discussions have considered the potential of network control theory for assessing psychological interventions^33^, with use-cases encompassing momentary experience quantification, cognitive behavior therapy, and mental disorder structuring^24^. Despite these optimistic developments, there remains a shortage of empirical testing of these theories. This deficiency is significant since while numerous models could theoretically 'explain' behavior, effective and predictable intervention requires a model that aligns with the system's inherent dynamics, namely the psychological construct. The "good regulator" theorem^34^ underscores this point, insisting that a successful regulator of a dynamical system must embody an accurate model of that system. Therefore, verifying a mathematical framework's ability to predict and guide interventions becomes a pivotal benchmark for model credibility. Given the current dearth of formal theories in psychology that endorse the use of dynamical systems theory, the urgency of this empirical validation is heightened.

This study seeks to tackle this challenge by evaluating the efficacy of network control theory in psychological perturbations through a representative experiment designed to alter attitudes towards meat consumption (see Fig. 1A). Pertinent to our objectives in this paper, network models have previously been used to study attitudes and are shown to be psychometrically realistic formalizations^4,10,35^. In this experiment, thirty healthy participants are asked about their attitudes toward eating meat using an 11-item questionnaire and are then subjected to 11 psychological interventions that aim to change their attitude on each item separately (see Methods for details). After an intervention, one intervention per item, participants are asked again the same 11 questions. Here, we build dynamic network models of the experiment and aim to predict the item-wise effect of the psychological intervention for each participant. Furthermore, based on fundamental results in control theory that relate the required energy for control to the intervention outcome, we hypothesize that the success of the interventions (i.e., sensitivity) is negatively associated with the psychological energy barrier (i.e., control theoretic measure of intervention energy) that is further dictated by the interactions between the response to the 11 questions.

## Results

### Efficiency of the psychological intervention

**Figure 2:**
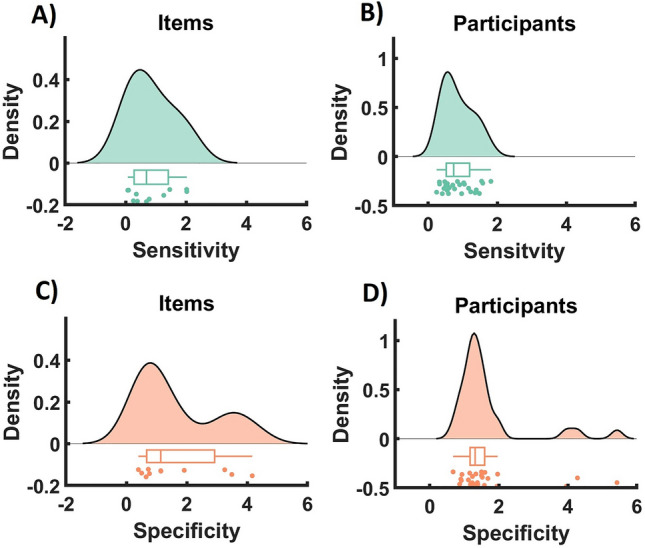
Sensitivity and specificity of interventions across items and participants. (**A**, **B**) Sensitivity is defined by the normalized changes in the responses (Methods, Eq. 2). Positive values indicate a change in the expected direction i.e., when the intervention was meant to reduce the rating, the subsequent rating after the intervention was indeed reduced and when the intervention was meant to increase the rating, the subsequent rating was indeed increased. (**C**, **D**) Specificity is defined by the relative absolute change of the intervened item compared to the average change of all the other items (Methods, Eq. 3). All specificity values are positive and higher values (of more than 1) indicating that perturbed items change more than the average of the other 10 items.

Figure 2 depicts the sensitivity and specificity (Methods, Eqs. 1–3) across all eleven items, scenarios, and participants. Our results show that, on average, perturbation was sensitive and affected the responses in the desired direction (i.e., most values are positive). However, there is large variability across participants (0.86 ± 0.44) and items (0.86 ± 0.73; mean ± std). On the other hand, the specificity reveals a more complex structure. Specifically, for the variability across items, we observe that the data seem to show two different clusters indicating that the interventions have been more specific for some items compared to others (1.65 ± 1.36). Interestingly, and in contrast to the item level, the specificity of the interventions shows only one cluster on the participant level indicating comparable specificity of the interventions across participants. Furthermore, we observe that sensitivity and specificity show a narrower distribution across participants than items, suggesting that variations in the effectiveness of the intervention are more comparable across participants than items.

### Network properties of intervention effects

A fundamental result in control theory relates the topological properties of the network of item-wise interactions ($A$, Eq. 4) to the effect of interventions applied to that item. Specifically, average and modal controllability measure the general ability of one variable to influence the value of all other variables. Therefore, interventions targeting nodes with higher absolute average or modal controllability should, on average, prove more effective. Average controllability pertains to the overall response within the system following a perturbation to the related node. Consequently, we posit a positive correlation between an item's average controllability and the intervention's sensitivity across participants and items. This means that as the average controllability increases, the sensitivity to intervention also typically increases. In contrast, modal controllability measures the capacity to govern fast modes, also known as difficult-to-reach states^36^. It is often inversely associated with average controllability^36^, leading us to hypothesize a negative relationship with sensitivity. This means that higher modal controllability might be associated with less sensitivity in the system. Specificity, on the other hand, is concerned with how effective an intervention is relative to the average of all interventions. Therefore, we anticipate a positive relationship with average controllability and a negative relationship with modal controllability. In both instances, we expect these relationships not to be stronger than that with sensitivity. We estimated the controllability metrics once based on the individual subjective perceived causal networks (i.e., self-reconstructed networks, see Methods) and once based on a data-driven generative model (see Methods for details) and assessed the correlation with the measures of sensitivity and specificity. Importantly, to avoid statistical biases due to the non-normal distribution of sensitivity and specificity metrics (Fig. 2)^37^, we use the rank correlation between controllability and perturbation effects. Our results (see Table 1) show that in both network models, average controllability correlates positively and modal controllability correlates negatively with sensitivity. We observe similar relation to specificity. Noteworthy, the size of the relations (r-values) is lower for the self-constructed networks than for the generative model and they do not reach statistical significance, but they have the same signs as those based on data-driven models.

**Table 1:** Kendal rank correlation between specificity/sensitivity and controllability measures (mean ± standard deviation across participants) with the number of significant tests shown in parentheses (n = 28 participants).

	Self-constructed Networks		Model	
	AC	MC	AC	MC
Sensitivity	0.07 ± 0.19 (n.s.)	− 0.07 ± 0.17 (n.s.)	0.20 ± 0.27 (3**)	− 0.19 ± 0.28 (4***)
Specificity	0.05 ± 0.22 (n.s.)	− 0.03 ± 0.22 (n.s.)	0.18 ± 0.24 (3***)	− 0.18 ± 0.24 (2***)

Group-level significance was assessed using Fisher’s Method^38^ (α < 0.05. Significant values are denoted with **P < 0.01 and ***P < 0.001.

### A mechanistic interpretation of intervention success

Having established that the network control theoretic metrics (i.e., average and modal controllability) contain meaningful information about the intervention effect, here we asked if the intervention success relates to the network structure. We base our hypothesis on the results from control theory that relates intervention success (in terms of sensitivity and specificity) to the intervention energy (i.e., effort, possibility) that can be exerted from an item. In our data, since we have no objective metric of the intervention’s actual energy (i.e. if some scenarios are fundamentally more powerful or more effective than others), we assume comparable intervention energy for all interventions (i.e., all scenarios) and thus hypothesize that the sensitivity should be smaller if the minimum amount of required intervention energy (i.e., the energy needed basing on the networks) is larger (for details see Methods, Eqs. 7–9). To do so, we estimated the association between the theoretically required energy and the sensitivity of the intervention (see Methods). Our results show that the sensitivity of the intervention (i.e., the extent to which the intervention works in the desired direction) is negatively associated with the theoretical energy (r-value = -0.22 ± 0.25; Fisher’s Method group level p-value = 7.32e-04) for the generative model. For the self-constructed networks, we also show statistically significant relation between sensitivity and energy (r-value = -0.13 ± 0. 25; Fisher’s Method group level p-value = 0.03) although the size of effect is smaller. Additionally, we find that the energy is negatively related to the specificity in both models, but the size of the effect is larger for the data-driven model (r = -0.16 ± 0.28; Fisher’s Method group-level p-value = 0.005; and r = -0.13 ± 0.16, Fisher’s Method group-level p-value = 0.05).

Finally, we tested if the model used in previous sections can predict the effect of interventions for every single item for every subject. Thereby, we simulated the model (Methods, Eq. 4) once based on the self-constructed and once based on the generative networks. We estimated prediction accuracy (ACC) in terms of the correlation between the predicted and observed responses after the intervention. Our results (Fig. 4) show that only the model correctly predicts the intervention effect (responses after the interventions); r = 0.84 ± 0.15[model]; and r = -0.04 ± 0.32 [self-constructed networks]).

## Discussion

Psychological interventions—including behavioral and cognitive therapies—are strategies aimed at triggering meaningful shifts in human emotions, responses, and behaviors. Despite an extensive body of research addressing a wide array of these interventions and their effects on human experiences, a comprehensive, systematic framework for evaluating these interventions has remained largely elusive^39^. Recognizing the success of network representations in encapsulating various psychological phenomena, we employ network control theory as a tool to quantitatively examine the impact of interventions. Originating from engineering, network control theory offers a robust approach for studying the changes in networked systems, making it a promising foundation for uncovering the mechanisms driving psychological interventions^6,15,19,22^. Despite theoretical discourse advocating its usage for bolstering our understanding of psychological constructs and interventions^24^, empirical studies testing its validity remain scarce.

**Figure 3:**
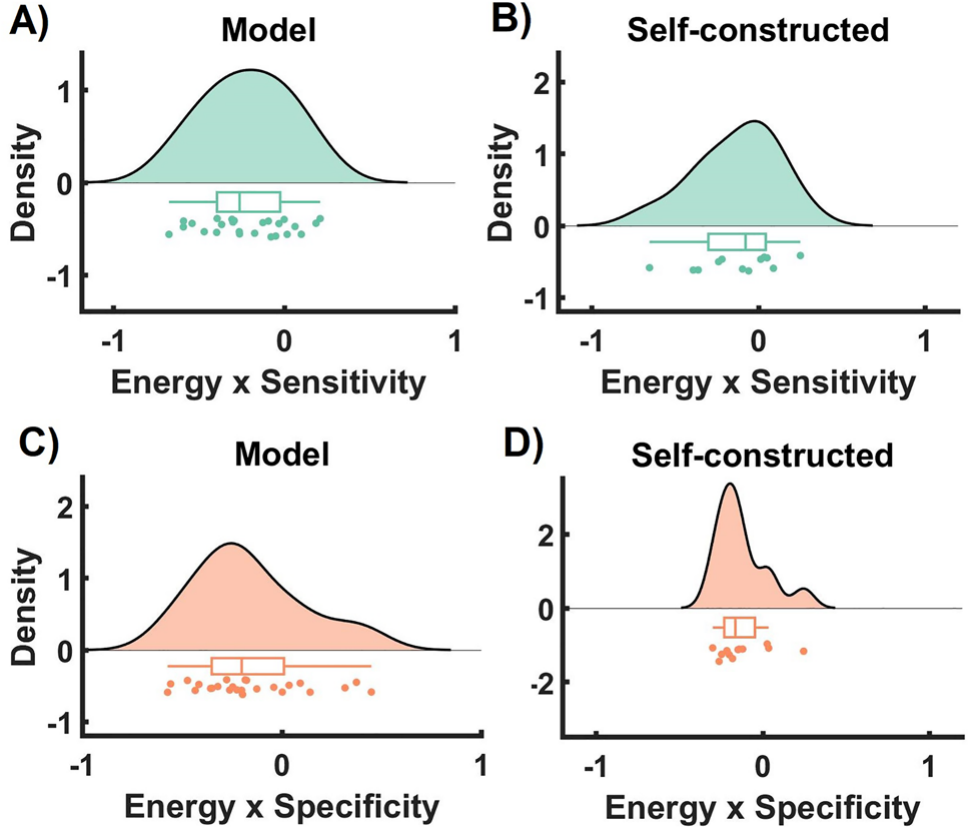
Network structure determines the success of psychological interventions. (**A**, **B**) Sensitivity is negatively associated with the network control theoretic estimation of the required energy to change the items only based on the generative model. (**C**, **D**) For both models (data-driven generative model as well as self-constructed networks), specificity is negatively related to the required energy but to a lesser extent compared to sensitivity.

**Figure 4:**
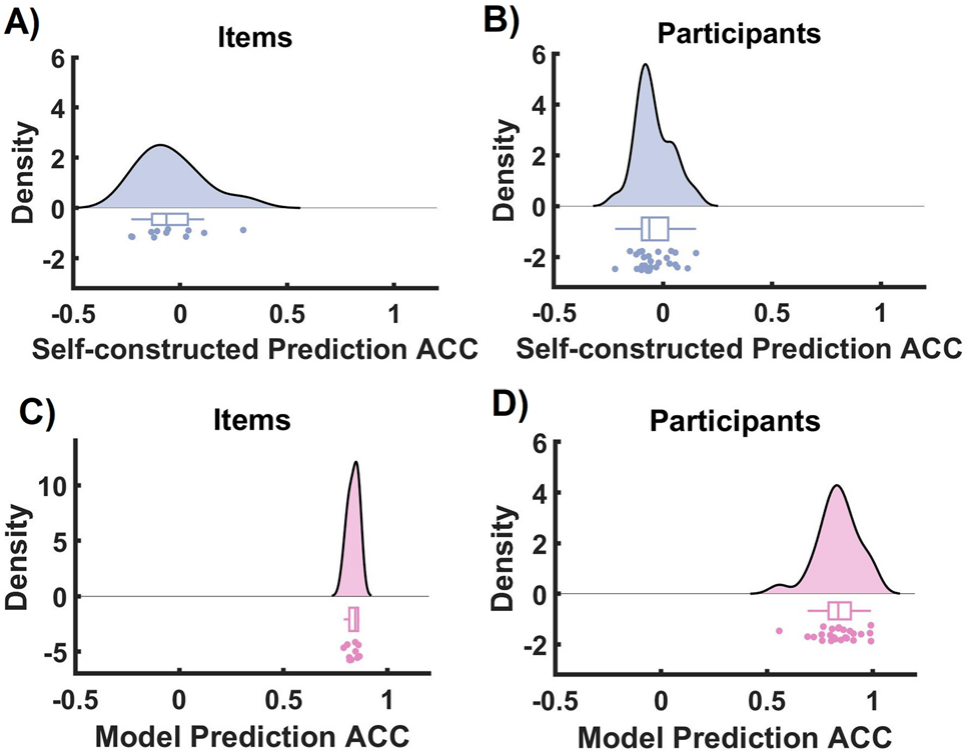
The generative model of psychological intervention accurately predicts the responses to the intervention. (**A**, **B**) Correlations between the predicted and actual responses averaged over items and participants for self-constructed networks. (**C**, **D**) Correlations between the predicted and actual responses averaged over items and participants for the generative model.

A pertinent example underpinning our study is a proof-of-concept psychological intervention task that aimed to modify attitudes towards meat consumption. We showed that the models provided by network control theory can predict the results of intervention on the level of the individual (Fig. 4), offer a mechanical account of why and how some of the interventions worked better than others (Fig. 3), and finally, used the model to show how sensitivity and specificity of the intervention relate to the network structure (Table 1). This work adds to the limited yet growing body of empirical evidence supporting the practical application of network control theory in psychological intervention studies.

### Conceptualization of psychological interventions through network control theory

The starting point to use network control theory to conceptualize and study psychological intervention is to build a mathematical model, specifically a dynamic system^11,24^. This system outlines the interrelations between the psychological variables involved in the construct under investigation—for instance, the responses to an 11-question survey—and the corresponding intervention, such as attitude-shifting scenarios. In the simplest case, the formulation presumes a linear association between these variables and an additive intervention effect, which depends on how each psychological variable in the system is impacted by the intervention (Eq. 4, Methods). The derivation process essentially hinges on estimating two sets of parameters: a matrix representing the relationships among the psychological variables, and a second matrix that connects the intervention to these variables. With these two sets of parameters established (matrices A and B in Eq. 4, Methods), network control theory offers a set of mathematically grounded estimations for the potential impact of any intervention on the psychological variables. Such an intervention could take the form of a one-time perturbation, like the one utilized in this study, or a series of successive perturbations. Importantly, the context-independent nature of network control theory's outcomes makes it a versatile framework for investigating psychological interventions across various contexts.

Importantly, even though interventions in our study were closely tied to the psychological variables (with a distinct intervention for each question in the 11-item questionnaire), this is not a mandatory criterion. The interaction between any given intervention and its effect on the psychological construct of interest can be captured using the same mathematical expression, merely adjusting the estimated parameters (matrices A and B) based on the collected data^40,41^. In essence, the overarching framework of network control theory is versatile enough to encompass the combined impacts of various intervention types, whether they are instruction-based as in our study, stimuli used in a priming task, financial incentives, and so on. These can be conceptualized as singular or recurring perturbations on targeted variables, like the 11-item questionnaire in our work, or clinical symptoms of conditions such as major depression. Once the dynamical system is defined—highlighting the specific psychological variables and interventions—and its parameters estimated (primarily matrices A and B in the linear scenario), one can gauge the intervention's influence on the psychological variables. This enables us to forecast outcomes under new conditions and potentially design more efficient interventions.

In our study, we developed two linear dynamical systems to analyze attitude intervention. In the first approach, the system was designed based on the causal interactions perceived by the participants (self-constructed networks, Matrix A) and the supposition that each intervention affects precisely one attitude aspect (Matrix B). Conversely, the second approach entailed a data-driven methodology where we made no assumptions about matrices A and B and instead derived them from the data. Over a range of metrics (Figs. 2–4), we found that the data-driven model is, by a large margin, superior to the model based on self-constructed networks. From a control theoretical perspective, this observation suggests that the data-driven model we obtained here is a plausible internal model of the system we studied but the one based on self-constructed networks is not. We notice that this result corroborates the theoretical findings calling for mathematical models of psychological behavior^32,39,42^: In the absence of models (for exceptions see Robinough et al.^25^), the generative models, for the time being at least^42,43^, must be built based on data-driven approaches.

### Methodological considerations

In general, a dynamical system can become extremely complex with many nonlinearities^44^. However, many examples across a wide range of applications in physics, engineering, and neurosciences have shown that a linear model (such that the one in Eq. 4, Methods) can be sufficient to explain most phenomena at least in close vicinity to the initial values^11,45^. This significantly simplifies the analytical treatment of the phenomenon (here psychological intervention) and provides a large repertoire of results that would all follow from the generative model. By employing a linear dynamical model of the intervention, it becomes possible to estimate the energy needed (intervention power) to modify the state variables (i.e., the psychological parameters under study). Additionally, one can determine the relative average significance of each variable in influencing the others. Most importantly, this approach enables the design of interventions that are optimal in terms of required energy, deviation from initial values, or time constraints^11^.

A crucial consideration in our study is the estimation of the model parameters. There is a rising interest in data-driven methodologies for inferring data-driven models in psychology. In this paper, alongside a self-constructed model grounded in the perceived causal relations reported by participants, we employed a method based on the dynamic mode decomposition^40^ (see Methods). Although beyond the scope of this paper, it would be intriguing to investigate whether alternative network identification tools might enhance our findings. Techniques such as sparse identification of nonlinear dynamics (SINDy)^46^, which has proven highly successful in various key fields including neuroscience^41,45,47^ as well as methods more frequently used in the psychological literature such as Gaussian graphical models^48^ or Bayesian network model^49^ could enhance our results (for a tutorial see Epskamp et al.^43^ and for critiques see^50^). These techniques exhibit several fundamental differences in both the parameters they estimate and the methods they employ to calculate those estimates^51^. For example, while methods like DMDc and SINDy concurrently estimate the full model, including both matrices *A* and *B*, approaches rooted in Bayesian estimation typically only provide an estimation for matrix *A*. In these cases, matrix *B* must either be defined based on the specific experimental conditions or estimated as a separate process. This distinction highlights the inherent differences in approach and underscores the need for careful consideration in selecting the appropriate method for a given analysis or application. This insight is particularly significant in shaping interventions based on a mathematical model. While a large number of, sometimes conflicting^52^, data-driven models could equally well describe a psychological phenomenon based on correlational studies^53^ (i.e., mathematical equations that describe the observed data in terms of e.g. correlations, see Methods for formal definitions), models that are mechanistically grounded (i.e., have a working model of the internal dynamics) are better equipped to inform us about possible interventions^54,55^. In this study, we assessed our methodology not only by comparing the model's predictions to the actual observed data but also by analyzing the theoretical aspects of the model, particularly the relationship between the intervention energy estimation and the analysis's sensitivity. While this approach lends substantial support to the models, we believe that the validation of the models requires further exploration and testing to ensure their robustness and applicability in various contexts. One approach to achieve this involves the application of control-theoretic intervention strategies to known underlying dynamics through simulation studies (as seen in the work by Lunansky et al.^9^). However, experimental work is also essential to define and establish the applicability of these methodologies (see Stocker et al.^56^ for an example of fundamental limitation in simulation studies), ensuring that they are not only theoretically sound but also practically useful and effective in various contexts.

### Limitations and future directions

Finally, we mention three major limitations of our approach and the representative example we used in this paper. First, in most cases, a therapeutic or preventive intervention does not happen in one step and encompasses multiple repetitions. Examples include psychotherapy, meditations, medication, neural stimulation, neurofeedback, physical and activity therapy, and psychological education^57^. Also, the use of hypothetical scenarios as intervening methodology is questionable. If and how our methodology would explain the effects of continued intervention should be addressed in further research. Related, the effect of an intervention is time-dependent. For instance, the effect of psychological priming is known to be mostly observable for a few minutes. In contrast, research on neurofeedback training and psychotherapy shows long-lasting effects. How and if such temporal variation can be included in the methodology presented in this paper must be further investigated.

Lastly, it is important to mention that despite the encouraging results presented in this manuscript, our study employed a small sample size and was specifically designed to accommodate network models. Therefore, this should be considered a proof-of-concept study. In essence, control theory has a broad range of applications, even without a distinct intervention in place (e.g., Jamalabadi et al.^58^). The question of whether our findings can be generalized to other contexts, such as those with a more diverse sample or where the intervention cannot be linked to specific nodes, thus necessitating more intricate data-driven methodologies, remains a topic for future investigation.

## Conclusion

In a variety of psychological subfields, networks have been proven to provide valuable in- and hind-sight into psychological behavior. In this paper, we demonstrated how such networks may be evaluated using network control theory, which is the engineering study of networks under intervention. In a representative case of attitude transformation, we demonstrated that the effects of the psychological intervention are heavily related to predictions provided by known control theory results. We also compared the performance of data-driven generative models to that of self-constructed network models and found that data-driven models provide a more accurate depiction of the intervention effect. In sum, network control theory may offer a formal theory to assess the (network-dependent) effects of psychological interventions and guide the construction of interventions.

## Methods

### Dataset

We use a publicly available and freely downloadable dataset, published in 2018 under the Journal of Open Psychology Data^35^. In short, thirty participants with ages ranging from 19 to 57 (median age 20 ± 9 years) were asked about their attitude toward eating meat (11 questions). The responses would be one of 6 possibilities between “completely disagree” and “completely agree”. The participants were then asked to contemplate 11 hypothetical scenarios one by one, corresponding to the 11 items in the questionnaire, which were designed to alter their opinion on each of the items (the list of questions and the scenarios are publicly available at http://osf.io/8tm5f). For instance, if a participant had a negative opinion on the morality of eating meat (i.e. initial response between 1–3), the participants were prompted to imagine that morality is only defined for humans and not necessarily for animals. After each perturbation, the participants were then asked the same 11 questions and were further asked about their perceived the causal relation between the perturbed item and the other items in the questionnaire. The participants had to draw these relations in an empty network (see Fig. 1). In this paper, we used the answers to the 11 questions as the state variables in our models and the prompts are considered as perturbations since they are designed to change the psychological state that described the attitudes towards eating meat. Further, we use the subjectively perceived causal relations which have been drawn by the participants to build dynamical systems (see sections "Derivation of networks" and "Network control theory and the effect of psychological perturbation").

### Quantification of the effect of perturbation

To quantify if and how the perturbations change participants’ responses, we define the perturbation effect ($e$) as the normalized difference between responses before and after perturbation for each subject and each scenario separately. We parametrize this effect further using two measures. First, we define the sensitivity of perturbation ($se$) as the signed net effect of the perturbation effect. That is, if the perturbation is meant to increase the value of the responses to a given question ($g = 1$; i.e., make the participants agree more with that question), sensitivity is estimated as the perturbation effect. Otherwise, that is, if the perturbation is meant to decrease the value ($g = - 1$), the sensitivity is defined as the negation of the perturbation effect. This way, a positive and large sensitivity signals a successful perturbation. Second, we define specificity ($sp$) as the efficiency of the perturbation in changing the value of the response to the certain question for which the perturbation is designed. That is, if the perturbation works (high sensitivity) for more than one question, then the specificity is low. Equations 1–3 summarize these definitions mathematically where $r_{ij } \in \left\{ {1:6} \right\}$ represents responses of $i^{th}$ subject to $j^{th}$ question, where $i \in \left\{ {1:30} \right\}$ represent the participants, $j \in \left\{ {1:11} \right\}$ represent the items (see Fig. 1), and $r_{i0}$ refers to the baseline response before the perturbations.

1 $$e_{ij} = \frac{{r_{ij} - r_{i0} }}{{r_{i0} }}$$

2 $$se_{ij} = e_{ij} \times g_{ij}$$

3 $$sp_{ij} = \frac{{\left\|e_{ij}\right\| }}{{\left( {\mathop \sum \nolimits_{k \ne j} \left\|e_{ik}\right\| } \right)/10}}$$

### Network control theory and the effect of psychological perturbation

Following previous work^15,22^, we assume the psychological behavior to follow a noise-free linear time-invariant model given by

4 $$x\left( {k + 1} \right) = Ax\left( k \right) + Bu\left( k \right)$$

where $x\left( k \right) \in D^{11} ,D = \left\{ {0,1,2,3,4,5,6} \right\}$ defines the attitude towards meat at time $k$ (also called the state), $A$ represents the interaction matrix (i.e., the networks and $B$ is the input matrix that specifies how the intervention affects $x$ (see section "Derivation of networks" for the estimation procedure), and $u\left( k \right)$ corresponds to the intervention parameters (for details see section "Dataset")^40^. Importantly, following previous work^15,26^, we assume that $A$ remains constant after intervention that we estimated once based on a data-driven methodology (see section "Derivation of networks") and once based on the subjectively perceived causal relation (see section "Dataset").

Based on this equation, we can compute the following metrics:

1. *Controllability metrics* Within the domain of network control, controllability metrics pertain to the characteristics of network nodes that enable them to direct the functional dynamics of the network when subject to perturbations. Specifically, these metrics provide an estimation of the extent to which the values of other nodes would be affected if a particular node experiences an external or internal stimulation. Consequently, these metrics serve as a means to evaluate the efficacy of interventions, measuring their potential impact and are naturally sensitive to diverse metrics that define the effectiveness of interventions. The literature has proposed a wide array of controllability metrics, each possessing applicability in specific contexts. However, two metrics, namely average controllability and modal controllability, have garnered particular attention due to their beneficial mathematical properties and demonstrated sensitivity to various functional properties^45^.
  Conceptually, average controllability is associated with the averaged interconnections between nodes, wherein nodes exhibiting higher average controllability are those for which interventions result in more pronounced changes around their current values. In contrast, modal controllability relates to the temporal modes of network changes following interventions on specific nodes. Statistically, average and modal controllability display a negative association^36^.
  In this paper, we focus on examining the state variables within the network, which correspond to the responses provided for 11 questions related to attitudes towards meat consumption (refer to section "Dataset"). The central hypothesis is that interventions targeting nodes with higher average and modal controllability are anticipated to yield a more substantial impact on the overall responses within the network, on average. In other words, by identifying nodes with elevated average and modal controllability, interventions can be strategically directed towards these influential nodes. Consequently, it is expected that these interventions will result in more significant changes in the responses across the network, given the heightened ability of these influential nodes to drive alterations in the overall system.
  Mathematically, the average controllability (AC) of node $j$ is defined as:
  5 $$AC_{j} = trace\left( {\mathop \sum \limits_{i = 0}^{\infty } A^{i} B_{j} B_{j}^{T} \left( {A^{T} } \right)^{i} } \right)$$
  where $A$ is the network under study and $B_{j}$ the $j^{th}$ canonical vector. Modal controllability (MC) is calculated by:
  6 $$MC_{j} = \mathop \sum \limits_{i}^{11} \left[ {1 - \xi_{i}^{2} \left( A \right)} \right]v_{ji}^{2}$$
  where $\xi_{i}$ and $v_{ji}$ are the eigenvalues and eigenvectors of $A$.
2. *Control energy* In the field of network control, control energy serves as a quantitative measure of the effort required to manipulate the collective state of a system, as represented by Eq. 4. Within the scope of the specific intervention experiment examined in this paper, control energy represents the combined strength of the employed scenarios aimed at inducing changes in the system's responses. As a result, interventions characterized by lower control energy are anticipated to bring about more substantial alterations, thus demonstrating heightened sensitivity.
  To provide further clarification, interventions with lower control energy necessitate less exertion or intervention power to achieve significant changes in the system's responses. Consequently, these interventions are expected to have a greater impact and exhibit enhanced sensitivity, as they possess inherent efficiency in instigating significant modifications in the overall network dynamics. It is important to note that control energy is defined with respect to the transition between two states, specifically the responses to the 11 questions before and after the intervention. It relates to the overall structure of the network and does not pertain to individual nodes, unlike controllability metrics, which assess the influence of each individual node in the system.
  We computed the energy required to move from $x_{0} = x\left( {k = 0} \right)$ to $x_{T} = x\left( {k = K} \right)$ based on perturbation of the $i^{th}$ question as follows according to the quadratic control function that is the most widely used formalization in literature^59,60^:
  7 $$E = u^{T} u$$
  where $u$ is the solution to the optimal control problem as in^26,61^.
  8 $$\mathop {\min }\limits_{u} \mathop \sum \limits_{0}^{T} [\left( {x_{K} - x\left( k \right)} \right)^{T} \left( {X_{K} - x\left( k \right)} \right) + \rho u\left( k \right)^{T} u\left( k \right)],$$
  9 $$s.t.x\left( {k + 1} \right) = Ax\left( k \right) + Bu\left( k \right),x\left( 0 \right) = x_{0} \;{\text{and}}\;x\left( T \right) = x_{T}$$
  where $K$ and $\rho$ are free parameters quantifying the time to reach from $x_{0}$ to $x_{T}$ and the relative importance of cost terms in Eq. (5). Following^62,63^, we define K $= 1$ and $\rho = 1$. To solve the optimal control Eqs. (5) and (6), we use a customized version of the code that is used elsewhere to study the brain as well as psychological dynamics^15,26,61^.

### Derivation of networks

In this manuscript, we build the networks (i.e., $A$ and $B$ in Eq. 4) in two ways.

- *Self-reconstructed networks* In these models, for each participant, $A_{11 \times 11}$ is defined to be equal to the individual perceived psychological interaction networks that each participant drew during the experiments (see section "Dataset" for a short and the original publication^35^ for a detailed description). In all cases, $U_{11 \times 1}$ (see Eq. 4), is equal to a vector where all elements are zero except for the $i^{th}$ element (corresponding to the $i^{th}$ intervention, $i \in \left\{ {1,2, \ldots ,11} \right\}$), which is either $+ 1$ or $- 1$ depending on the intention of the intervention (i.e., either to make the participants agree or to disagree with an item in the questionnaire ). Following the logic of the experiment, $B_{11 \times 11}$ is then set to be equal to a matrix where all elements all zeros except for $B\left( {i,i} \right)$ which is equal to $+ 1$.
- *Data-driven Models* In these models, we estimated $A_{11 \times 11}$ and $B_{11 \times 11}$ based on the data. Specifically, we used Dynamic Mode Decomposition with Control (DMDc^40^) which is one of the most successful and robust data-algorithms in the literature and has several theoretical advantages that makes it interesting for our study^64^: not only is it suitable for sparse data, but it can also be employed in nonlinear systems, thanks to its connections to the Koopman operator. In the most straightforward implementation which we used in this paper (see Proctor^40^ for methodological considerations), defining $X_{1} = \left[ {x_{1} x_{2} \ldots x_{m - 1} } \right]$, $X_{2} = \left[ {x_{2} x_{3} \ldots x_{m} } \right]$ $U = \left[ {u_{1} , u_{2} \ldots u_{m - 1} } \right]$ where $x_{i} = x\left( i \right)$ and $u_{1} = u\left( k \right)$, we can rewrite Eq. 4 and thus solve for A and B simultaneously as follows:
  10 $$X_{2} = \left[ {A B} \right]\left[ {\begin{array}{*{20}c} {X_{1} } \\ U \\ \end{array} } \right]$$
  11 $$\left[ {A B} \right] = X_{2} \left[ {\begin{array}{*{20}c} {X_{1} } \\ U \\ \end{array} } \right]^{\dag }$$
  where $\dag$ denotes Moore–Penrose pseudoinverse^61^. In this paper, we estimated $A$ and $B$ for individually each subject separately where $X_{1}$ was filled with the same initial state before the start of the interventions, $X_{2}$ with the recorded responses following 11 interventions. Additionally, we configured and $u_{11 \times 1} \left( i \right)$ such that all elements were set to zero, except for the specific entry corresponding to the active intervention, which was assigned a value of 1.

## Data Availability

The codes to replicate the simulations are publicly available at https://osf.io/r7xsz/. The data used in this study is publicly available at http://osf.io/8tm5f.
